# Non-invasive spinal vibration testing using ultrafast ultrasound imaging: A new way to measure spine function

**DOI:** 10.1038/s41598-018-27816-0

**Published:** 2018-06-25

**Authors:** Tarek Kaddoura, Anthony Au, Greg Kawchuk, Richard Uwiera, Richard Fox, Roger Zemp

**Affiliations:** 1 Department of Electrical and Computer Engineering, University of Alberta, Edmonton, Alberta Canada; 2 Department of Physical Therapy, University of Alberta, Edmonton, Alberta Canada; 3 Department of Surgery, University of Alberta, Edmonton, Alberta Canada; 4 Department of Agricultural, University of Alberta, Food and Nutritional Science, Edmonton, Alberta Canada

## Abstract

Ultrafast ultrasound imaging is used to capture driven spinal vibrations as a new method for non-invasive spinal testing in living subjects. Previously, it has been shown that accelerometer-based vibration testing in cadaveric models can reveal the presence, location and magnitude of spinal pathology. However, this process remains an invasive procedure as current non-invasive sensors are inadequate. In this paper, the ability of non-invasive ultrafast ultrasound to quantify *in vivo* vertebral vibration response across a broad range of frequencies (10–100Hz) in anesthetized pig models is investigated. Close agreement with invasive accelerometer measurements is achieved using the non-invasive ultrasound method, opening up unique opportunities to investigate spinal pathologies.

## Introduction

Globally, low back pain is the leading cause of years lived with disability^1^. As a result, the direct and indirect costs of low back pain are enormous adding to the significant morbidity caused by this chronic, recurrent condition. Unfortunately, only 10 percent of low back pain cases have an identifiable cause^2^. Without knowing its cause, the prevention, identification and treatment of low back pain is ineffective at best which perpetuates its impact on society.

While there are many ways to measure spinal function (e.g. range of motion), current limitations in diagnosing back pain are thought to arise largely from the failure of current tests to measure spinal functions that are linked to meaningful clinical outcomes. Similarly, current imaging techniques display static anatomy very well, but reveal little about the function of a mechanical structure like the spine.

Given the above, we have looked to examples of technologies able to evaluate the functional status of mechanical or geological targets. One such technology is non-destructive vibration testing which is used to identify the location, presence and quantity of oil deposits in seismic testing and structural flaws in aerospace and civil construction projects^3–8^.

With this approach, we have shown previously that when vibration is applied to cadaveric spines, the response of accelerometers implanted within lumbar vertebrae are unique and can be used to identify a range of simulated pathologies^9–11^. For example, the vibration spectrum approach was capable of differentiating vertebrae tied together (to reduce inter-vertebral motion) which simulated disk degeneration. Additionally, progressive levels of injury simulating increased vertebral motion (modeling, for example, herniated discs) could be detected from vibration signatures^9,10^. Unfortunately, this invasive approach has limited clinical application. In addition to model experiments, a previous clinical study using surface-based accelerometers was able to demonstrate that signals from different pathologies are unique in human twins^12^. In this study, five sets of discordant twins showed unique vibration signals in contrast to five sets of concordant twins, where signals overlapped. For example, pairs of healthy twins showed similar signals while twins with spine pathologies such as herniated discs, disc degeneration and hemangioma as validated by MRI, showed discordant vibration responses^12^. Despite the promise of this study, placement and replacement of surface sensors was found to be problematic due to potential contamination from peripheral tissues^13^.

Given the long-term goal to classify abnormal spinal vibrational modes with spinal pathologies, this paper explores the possibility that non-invasive, ultrafast ultrasound can be used to estimate spinal dynamics and that this approach is valid when compared to implantable accelerometers, the present gold-standard.

Ultrafast ultrasound methods have gained considerable attention in recent literature and use fast transmit sequences to enable imaging at thousands of frames per second^14^, in contrast to more traditional scanline imaging approaches which offer only realtime frame-rates. Ultrafast imaging approaches include plane-wave imaging, plane-wave compounding, diverging wave imaging, and fast synthetic aperture approaches. These ultrafast imaging approaches have primarily been used to image soft tissue displacements^15–17^, shear-waves^18–20^, and blood flow^21–24^ however, to our knowledge, these methods have not yet been applied to estimating vibrations in bone-structures *in vivo*. Lower-frame-rate ultrasound or Doppler ultrasound methods have been used to image bone-vibration structures such as loose hip-cups^25^, however, no validation was performed *in vivo* and these methods lack the ability to track fast motions over large fields-of-view.

M-mode ultrasound tracking of spinal vibrations were previously considered but because of significant 3D motion, results were poor. For example, M-mode interrogation locations shifted considerably over time during vibration, which resulted in poor displacement measurements. The proposed ultrafast imaging approach enables full-frame motion characterization, albeit with less accurate lateral displacement estimation.

Here we describe a series of experiments performed on an anesthetized pig model to test the hypothesis that plane-wave-based ultrafast ultrasound could achieve similar accuracy to the invasive accelerometer measurements. This paper represents a step towards a future non-invasive functional technique to differentiate spinal pathologies.

## Methods

**Figure 1:**
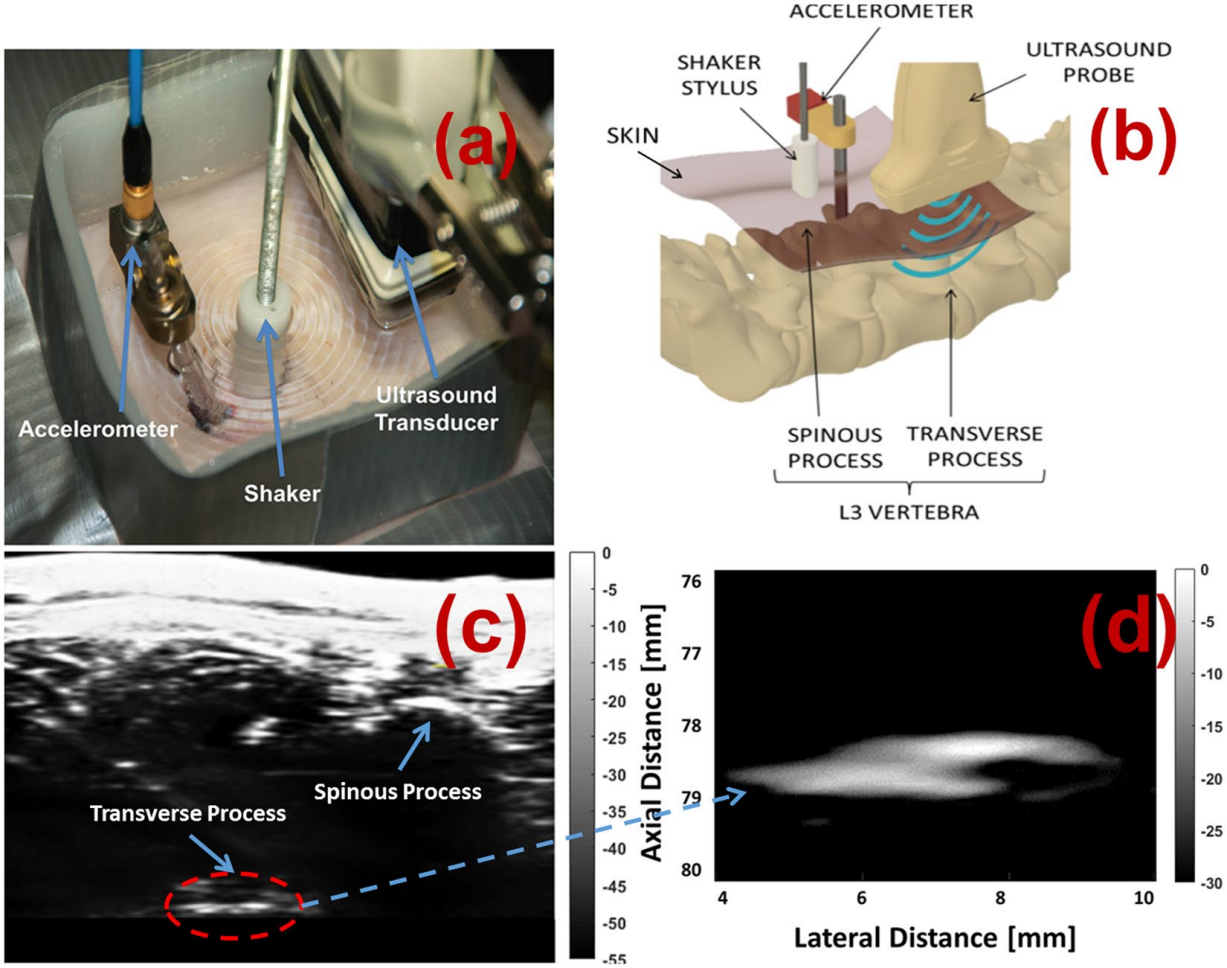
A picture and schematic of the experimental setup (**a**,**b**), a high quality ultrasound image of a spine imaged at 60 frames/sec (**c**), and an ultrafast ultrasound image of a transverse process imaged at 500 frames/sec (**d**). A bone pin is drilled into the left L4 vertebral body of the spine and then an accelerometer attached to the top of the bone pin for reference. A shaker stylus is attached to the top of the skin surface to provide vibration. An ultrasound transducer submerged in water is placed beside the setup on top of the skin close to the right L4 transverse process. Standing waves can be seen generated in the water on the top-left image due to a vibrational input.

Seven pigs (58–68 kg) were used in this experiment. All pigs were provided by the University of Alberta’s Swine Technology Research Centre. This protocol was approved by the University of Alberta Research Ethics Office (AUP00001898) and adhered to throughout the experiment. Vibration was provided to the L4 vertebra in each anesthetized animal via a computer-controlled stylus lowered on to the skin posterior to the L4 spinous process. The reference system consisted of a 3mm-diameter, 15cm-length bone pin drilled through the surface of the skin and into the left L4 vertebral body. A tri-axial accelerometer (PCB Piezotronics Inc. model 356A35) was then connected to the exposed pin and a ball joint used to orient the sensor to the global coordinate system. The experimental system consisted of an ultrasound transducer placed posterior to the right L4 transverse process to collect images of the vertebral position at 500 images/sec using plane wave imaging. Figure 1 shows (a) a picture of the experimental setup, (b) a schematic of the experimental setup, (c) a high quality ultrasound image of a spine imaged at 60 frames/sec, and (d) an ultrafast ultrasound image of a transverse process imaged at 500 frames/sec.

Data from the accelerometer was obtained using a VR9500 control system (Vibration Research Corp., Chicago, MI) connected to a computer with VibrationVIEW software. Vibration to the spine was provided using a ET-126 shaker (Labworks Inc., Costa Mesa, CA) using the same control system. Vibrations were applied with a target displacement range of 3 mm peak-to-peak for the lowest frequency case, down to 0.25 mm for the highest frequency case and for chirp vibration tests. Displacement tolerance was set to $\pm \mathrm{15 \% }$. Ultrasound data from the spine was acquired and processed using a programmable ultrasound system (Vantage 256, Verasonics, Redmond, WA) and a 5 MHz 128-element imaging transducer array (L7–4, Philips ATL, WA). RF channel data was acquired and RF beamformed images were reconstructed offline using dynamic receive beamforming algorithms.

For each pig, vibration was first applied with single-frequency sine waves ranging from 10–100 Hz in 10 Hz increments for 10 seconds each to test the single-frequency response. In each single-frequency sine test, we induced a breath-hold for around 4 seconds through the respirator used to control the animal’s breathing under anesthesia. This was done to ensure that the breathing pattern of the pig did not interfere with the resulting vibrational response. In addition to the single sine tests, two chirp vibration tests were performed to test the vibrational response across many frequencies in a single test. Chirps with frequencies of (1) 10–50 Hz, and (2) 50–100 Hz were used for these tests. For the chirp tests, it was not possible nor safe to perform breath holds for 10 seconds, and hence we opted to perform the chirp tests with the pigs breathing. Since the chirp tests were done with breathing, we evaluated whether the ultrasound imaging technique were able to detect vibrational patterns accurately under these conditions.

For each recording obtained, we performed the following pre-processing steps: (1) displacement of the spine as measured by ultrasound processing of beamformed RF images was calculated between image frames using an adaptive cross-correlation algorithm. This algorithm consists of a reference window as big as the image frame, and a rectangular sliding window manually specified to be centered on the transverse process. The sliding window was cross-correlated against the reference window to find the displacement shifts^26^, and then the sliding window is moved with the displacement shifts for the next frame. This allows the sliding window to always be centered on the spine target; (2) ultrasound displacement signals are then converted to acceleration using double differentiation using the “diff” function in MATLAB (The MathWorks Inc., Natick, MA); (3) ultrasound and accelerometer acceleration signals are then filtered using a 6th order Chebyshev bandpass filter between 0.5 times the lowest vibrational frequency and 1.5 times highest vibrational frequency to analyze only the portion of frequencies that are important for the specific test; (4) the accelerometer signal is then filtered using a 6th order Chebyshev Bandstop Filter at frequencies of 60 and 120 Hz to remove the 60 Hz electrical noise and its harmonics; and (5) filtered ultrasound and accelerometer acceleration time-series estimates are then aligned in time using a cross-correlation operation^27–29^.

After pre-processing, we compared the ultrasound and accelerometer acceleration signals using the following measures:

- Time Correlation: The Pearson correlation coefficient was calculated between the ultrasound and accelerometer acceleration signals in the time domain.
- Frequency Correlation: The Welch power-spectral-density (PSD) of each of the ultrasound and accelerometer acceleration signals was first calculated, then the Pearson correlation coefficient was calculated between the two PSDs.
- Magnitude Similarity: The root-mean-square (RMS) value for each of the ultrasound and accelerometer acceleration signals was calculated, then the relative error between the two RMS values was found using the following formula,

$$RM{S}_{Rel.Error}=\frac{|RM{S}_{Ultrasound}-RM{S}_{Accelerometer}|}{RM{S}_{Accelerometer}}$$

### Data availability

The raw ultrasound datasets analyzed during this study and their corresponding processed datasets are available from the corresponding author on request.

## *In Vivo* Results

Here we present our raw data for each of the seven pigs and for each individual frequency.

**Figure 2:**
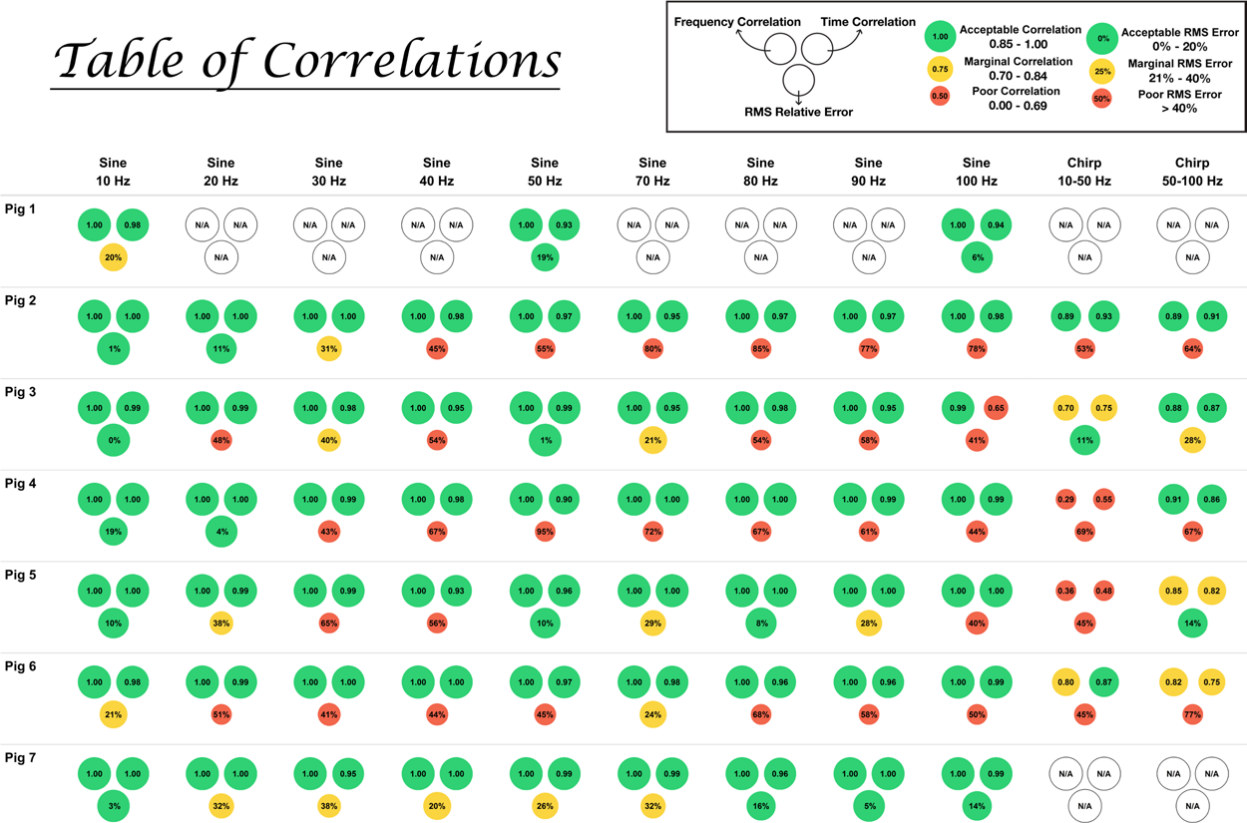
Frequency correlation, time correlation, and root-mean-square (RMS) relative error for all seven pigs across all tests. The size of each circle in the table represents the strength of the correlation with the exact measurements shown in center of the circle. Color-codes help show the strength of the results with green indicating an acceptable result, yellow indicating a marginal result, and red indicating a poor result.

Figure 2 shows a table of the computed frequency correlation, time correlation, and magnitude similarity for each pig and each frequency. The size of each circle in the table represents the strength of the correlation with the exact measurements shown in the center of the circle. The circles are color-coded for ease of visualization, with green indicating acceptable correlation, yellow indicating marginal correlation, and red indicating poor correlation. Furthermore, we defined (1) acceptable correlation as a Pearson correlation coefficient between 0.85 and 1, (2) marginal correlation as between 0.70 and 0.84, and (3) poor correlation as between 0 and 0.69. Similarly for relative RMS error, we defined (1) acceptable RMS error as a relative error of 0–20%, (2) marginal RMS error as 21–40%, and (3) poor RMS error as greater than 40%. From Fig. 2, we can see how most of our time and frequency correlations across all frequencies and all pigs are excellent. However, the magnitude correlations performed less well.

**Figure 3:**
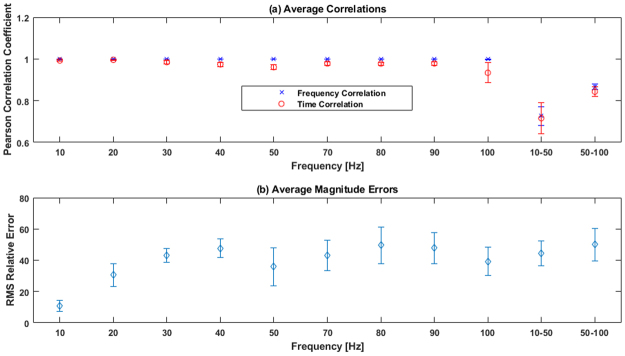
Averaged correlation and magnitude results. For each vibrational frequency, the Pearson correlation coefficient in the frequency-domain (top graph, blue marker) and the time-domain (top graph, red marker) was averaged across all seven pigs. Root-mean-square (RMS) relative errors are also averaged for each frequency (bottom graph). Error bars represent standard error.

In addition to referring to the data in Fig. 2 directly, we summarize all of our correlations and magnitude errors in Fig. 3. Figure 3 shows (a) time and frequency correlation averaged across all seven pigs for each vibrational frequency, and (b) RMS magnitude errors averaged across all seven pigs for each vibrational frequency.

From Fig. 3a, we can see how our time and frequency correlations were all very high (close to 1.0) for all our single sine tests. However for the chirp tests, we see a decrease in correlations down to 0.70. From Fig. 3b, we can see how the magnitude errors are not as good as our frequency and time correlations. We observed low relative errors of 1–20% for the lower frequencies (10 Hz–20 Hz), and then an average relative error of 40% for higher frequencies ($>$20 Hz). In addition, at higher frequencies the relative error is consistently around 40% and doesn’t vary much beyond that.

**Figure 4:**
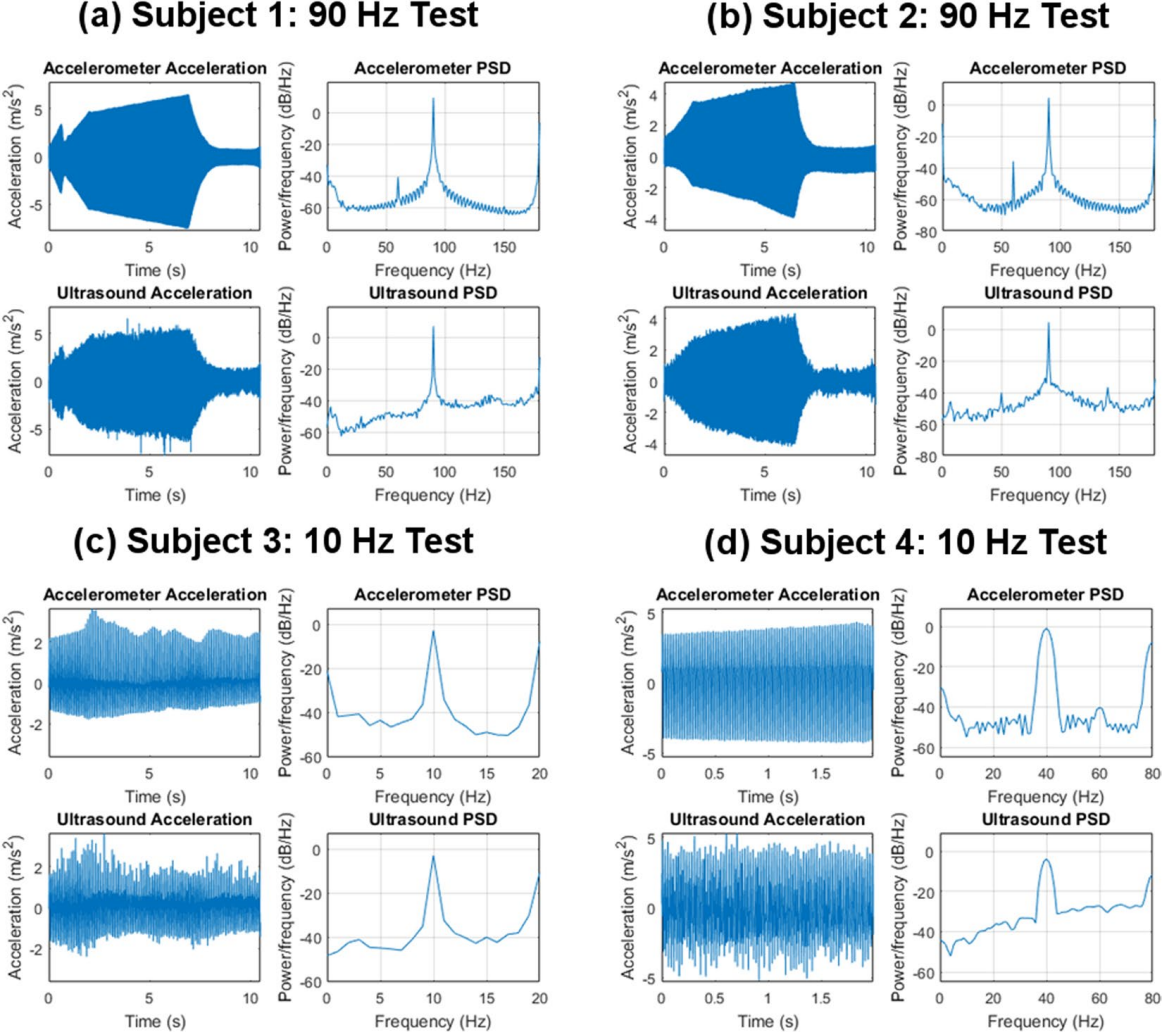
Selected samples showing comparisons between ultrasound acceleration and accelerometer acceleration recordings for single-sine tests. (**a**) a 90 Hz recording, (**b**) another 90 Hz recording, (**c**) a 10 Hz recording, and (**d**) a 40 Hz recording. The top of each graph shows the acceleration obtained from the accelerometer, and the bottom of each graph shows the acceleration obtained from ultrasound. The right of each graph shows the power-spectral-density of the acceleration signal on the left.

The majority of our ultrasound recordings were very clear and contributed to excellent correlations in our final results as shown in Fig. 2. Figure 4 shows a few select samples of ultrasound displacement recordings converted to acceleration that demonstrate the ability for ultrasound imaging to non-invasively generate vibrational graphs that are as clear and accurate as a gold standard accelerometer. Figure 4 shows (a) a 90 Hz recording, (b) another 90 Hz recording, (c) a 10 Hz recording, and (d) a 40 Hz recording.

**Figure 5:**
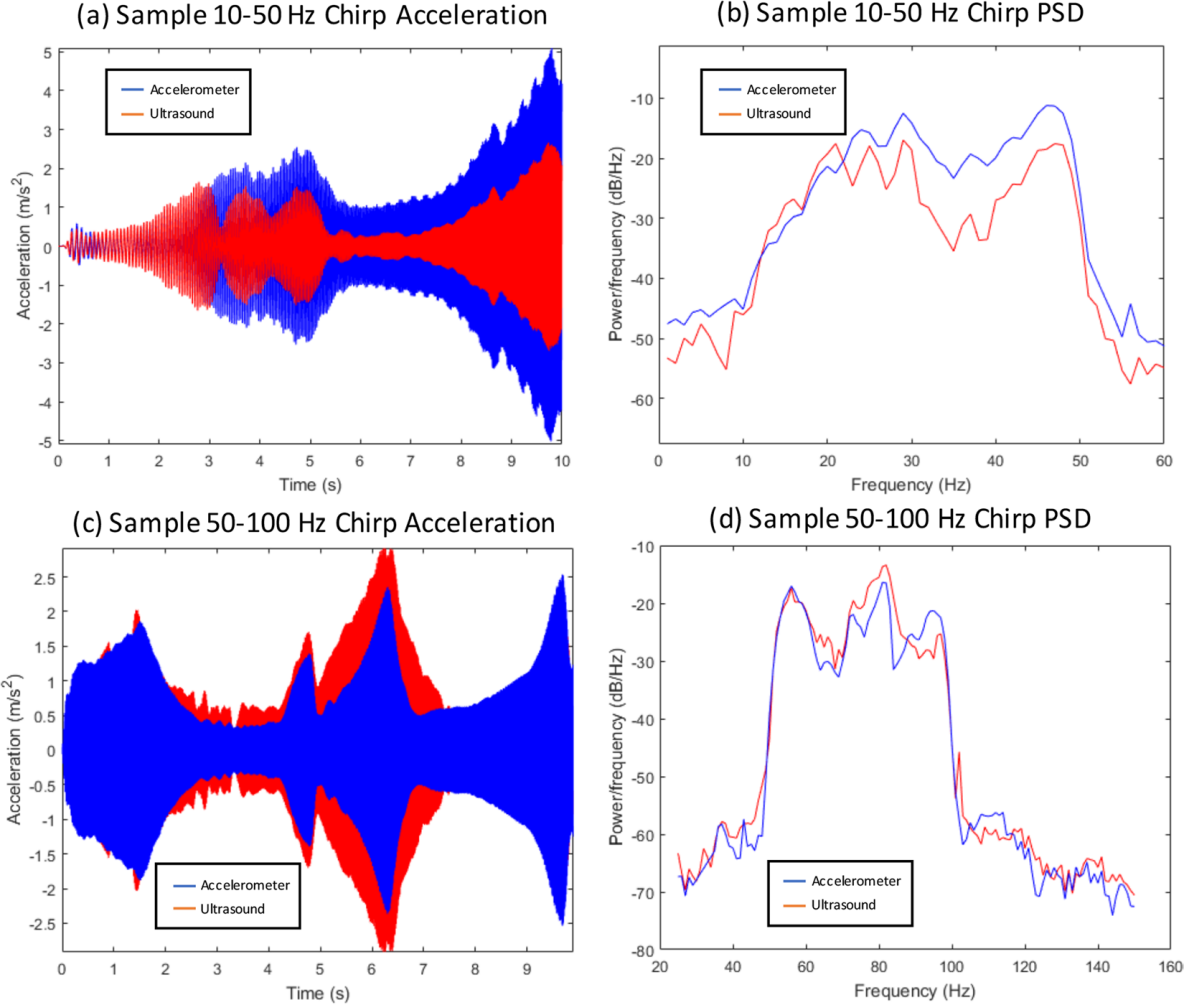
Selected samples showing comparisons between ultrasound acceleration and accelerometer acceleration results for chirp tests. The top row shows a 10–50 Hz chirp recording with the ultrasound and accelerometer results overlaid on top of each other. The bottom row shows a 50–100 Hz chirp recording. The left column of each row shows the time-domain of the acceleration signal, while the right column shows the power-spectral-density of the same signals.

Figure 5 also shows a couple of select chirp samples that achieved very high correlation results. We can visually see from these samples how there is very good agreement in both time and frequency.

## Discussion

Ultrafast ultrasound imaging allows us to image very fast events, potentially at thousands of frames per second. In this paper, we attempted to measure the vibrational responses of the spine in order to assess the feasibility of using ultrasound imaging for non-invasive quantification of spine vibration response. The imaging speeds of ultrafast ultrasound visualization are well-suited for measuring these fast events and the higher frame-rates of ultrafast imaging allow us to capture many samples for a single vibrational cycle. This allows for very good time resolution of our captured vibrational signals with sub-sampling of our signals for even greater accuracy in quantifying vibration responses.

While the driving force is sinusoidal, the actual forces transduced into the tissue will be asymmetric due to changing tissue coupling and changing ligamentous boundary conditions. Due to these asymmetrical forces we do not anticipate and do not see a purely sinusoidal vibration. Additionally, breathing motion introduces changes in coupling forces, which also distorts the sinusoidal response in Fig. 4.

For the single-sine tests, we see very good correlations in both frequency and time when using the Pearson correlation coefficient to compare the accelerometer results to the ultrasound results. In the frequency domain, all correlations for the single-sine tests exceed 0.98, while in the time domain, all correlations exceed 0.94. This indicates very good single-sine performance compared to the accelerometer. Hence, these results demonstrate that for simple single-sine vibrations, ultrasound imaging can be used non-invasively to obtain clear and accurate spine vibrational measurements compared to a gold-standard and invasive accelerometer. The accuracy of these results is visualized in Fig. 4 where we see very good agreement in both time and frequency between the ultrasound and the accelerometer.

For the more complex chirp vibration tests, we see on average worse performance compared to the single-sine tests. Since the chirp tests were performed without a breath-hold, it is expected that the performance will be worse than the single-sine tests due to the vibrational noise added from the internal body movements. For the 10–50 Hz chirp test, we found an average time-correlation of 0.75 and an average frequency correlation of 0.78. For the 50–100 Hz chirp test, we see an average time-correlation of 0.85 and an average frequency correlation of 0.88. While the correlations aren’t as good on average as the single-sine tests, we still had certain cases of chirp tests where the correlations were almost as good as the single-sine tests. For example, both chirp test correlations for Pig 2 exceeded 0.89 in both time and frequency domain, while at least one chirp test from Pigs 3, 4, and 6 exceeded 0.90. The strength of these correlations is shown graphically in Fig. 5. We can visually see how for these cases, there is very good agreement in both time and frequency. These specific results indicate that it is possible to obtain accurate chirp vibrational results using a non-invasive ultrasound system even when the subject is breathing.

While our results demonstrate very good time- and frequency-domain correlations, the root-mean-square (RMS) magnitude of our acceleration-converted ultrasound signals does not match the accelerometer’s results as well as our correlations. We observed magnitude errors between 1% to 45%, with lower frequencies having a lower magnitude error (1–20%) and higher frequencies and the chirp tests having a higher magnitude error (20–45%).

We attribute the high magnitude errors, the lower time-domain performance, and the worse chirp performance to multiple factors that are explained below.

First, we employed ultrafast ultrasound imaging, which provides very fast imaging speeds, but worse image quality compared to slower imaging methods. Due to time constraints related to anesthesia and system memory limitations in our experiment, we opted to use only one plane-wave angle to image the spine. This resulted in poor image quality where the spine target is visible but noisy. The poor image quality translated to a noisier ultrasound displacement signal compared to the accelerometer. Future work could also use coded excitation to improve signal-to-noise ratio. This noise is then further amplified by the double-differentiation operation to convert the displacement signal to acceleration. This can be mitigated in the future by using multiple coherently-compounded plane-wave angles to achieve better image quality at a reasonably high imaging speed^30^. With less noise in the image, the accuracy of the measurements should improve. Coherent compounding, however, may also be susceptible to significant motion artifacts owing to induced vibrations having velocities greater than 0.5 m/s.

Second, from our tri-axial accelerometer, we found that most of the vibrational movement of the spine is in the elevational and lateral directions. This was further confirmed in the ultrasound images by observing the spine target disappearing in one frame and then reappearing at the next frame. When the spine moves out-of-plane, it degrades the accuracy of the cross-correlation displacement estimate, and these errors propagate further down the signal and into the converted acceleration signal.

Third, it is very difficult to align the ultrasound transducer and the accelerometer to exactly the same imaging plane. This leaves room for small variations in the magnitude and shape of the signal relative to each other since they are no longer sampled from the same plane. This alignment issue coupled with the out-of-plane movements of the spine could negatively alter the time-correlation and magnitude results shown above.

The effect of these issues can be reduced by imaging a vibrational event multiple times and from multiple orientations to obtain a clearer and higher quality image that represents the true vibrational response of the spine. Multiple results from different orientations can be averaged and processed further to give a more robust 3-D visualization of the movement.

Other than cross-correlation displacement estimation, there are a wide variety of motion estimation techniques in ultrasound^31–33^. These include examples such as block matching, maximum-likelihood motion estimation, and intensity-independent feature tracking. In the future, different motion estimation techniques will be tested to try to improve displacement results.

Despite these limitations, non-invasive ultrasound imaging appears to perform well in quantifying vibrational time signatures and frequencies compared to the gold-standard invasive accelerometer. Hence, these results show that ultrafast ultrasound imaging is a very good candidate for assessing non-invasive spinal pathology. In the future, we will explore the ability of ultrasound to differentiate spinal vibrational signatures based on different spinal pathologies.

## Conclusion

Temporal and spectral signatures of driven spinal vibrations estimated from non-invasive ultrafast ultrasound are accurate when compared to an invasive gold-standard accelerometer in a study involving a cohort of 7 swine. Future work will focus on classification of different spinal pathologies by analyzing spine vibrational signatures from live subjects.
